# *In Vitro* and *in Vivo* Selection of Potentially Probiotic Lactobacilli From Nocellara del Belice Table Olives

**DOI:** 10.3389/fmicb.2018.00595

**Published:** 2018-03-28

**Authors:** Barbara Guantario, Paola Zinno, Emily Schifano, Marianna Roselli, Giuditta Perozzi, Claudio Palleschi, Daniela Uccelletti, Chiara Devirgiliis

**Affiliations:** ^1^Food & Nutrition Research Centre, Council for Agricultural Research and Economics, Rome, Italy; ^2^Department of Biology and Biotechnology “C. Darwin”, Sapienza University of Rome, Italy

**Keywords:** foodborne bacteria, fermented foods, nematode, autochtonous bacteria, plant food matrix

## Abstract

Table olives are increasingly recognized as a vehicle as well as a source of probiotic bacteria, especially those fermented with traditional procedures based on the activity of indigenous microbial consortia, originating from local environments. In the present study, we report characterization at the species level of 49 Lactic Acid Bacteria (LAB) strains deriving from Nocellara del Belice table olives fermented with the Spanish or Castelvetrano methods, recently isolated in our previous work. Ribosomal 16S DNA analysis allowed identification of 4 *Enterococcus gallinarum*, 3 *E. casseliflavus*, 14 *Leuconostoc mesenteroides*, 19 *Lactobacillus pentosus*, 7 *L. coryniformis*, and 2 *L. oligofermentans*. The *L. pentosus* and *L. coryniformis* strains were subjected to further screening to evaluate their probiotic potential, using a combination of *in vitro* and *in vivo* approaches. The majority of them showed high survival rates under *in vitro* simulated gastro-intestinal conditions, and positive antimicrobial activity against *Salmonella enterica* serovar Typhimurium, *Listeria monocytogenes* and enterotoxigenic *Escherichia coli* (ETEC) pathogens. Evaluation of antibiotic resistance to ampicillin, tetracycline, chloramphenicol, or erythromycin was also performed for all selected strains. Three *L. coryniformis* strains were selected as very good performers in the initial *in vitro* testing screens, they were antibiotic susceptible, as well as capable of inhibiting pathogen growth *in vitro*. Parallel screening employing the simplified model organism *Caenorhabditis elegans*, fed the *Lactobacillus* strains as a food source, revealed that one *L. pentosus* and one *L. coryniformis* strains significantly induced prolongevity effects and protection from pathogen-mediated infection. Moreover, both strains displayed adhesion to human intestinal epithelial Caco-2 cells and were able to outcompete foodborne pathogens for cell adhesion. Overall, these results are suggestive of beneficial features for novel LAB strains, which renders them promising candidates as starters for the manufacturing of fermented table olives with probiotic added value.

## Introduction

The most recent definition of “probiotic” was formulated by an expert consultation of international scientists working on behalf of FAO/WHO (Food and Agriculture Organization/World Health Organization), and refers to viable, non-pathogenic microorganisms (bacteria and/or yeast) that when ingested in adequate amounts, are able to reach and colonize the Gastro-Intestinal (GI) tract and to confer health benefits to the host (FAO/WHO, 2006). The main benefits associated with probiotic intake include gut health and immune modulation (Ritchie and Romanuk, 2012; Hill et al., 2014). In particular, probiotic consumption can influence the microbial composition and balance within the intestinal microbiota. Production of antimicrobial substances such as organic acids, hydrogen peroxide, antifungal peptides, and bacteriocins, contributes to decrease harmful microorganisms and promotes growth and stability of beneficial bacteria, such as lactobacilli and bifidobacteria (Magnusson and Schnürer, 2001; Baker et al., 2009; Abriouel et al., 2012; Amund, 2016; Hegarty et al., 2016). Probiotic capacity greatly varies among strains belonging to different genera and species. The most common bacterial strains employed as probiotics are found within LAB (Lactic Acid Bacteria) species belonging to the *Bifidobacterium, Lactobacillus*, and *Streptococcus* genera (Saulnier et al., 2009). Members of the *Lactobacillus* genus are especially relevant as foodborne probiotics because they can be exploited also from the technological viewpoint, as their metabolic properties lead to production of a wide spectrum of molecules conferring specific organoleptic quality to fermented products. Moreover, several lactobacilli are considered Generally Recognized as Safe (GRAS) and are largely used as starter and/or protective cultures in fermented vegetables, sausages, fish and dairy products (Giraffa et al., 2010; Garrigues et al., 2013; Montoro et al., 2016).

The growing demand for plant-based foods is presently driving selection of bacteria which are able to grow on fermentable vegetable sources (Granato et al., 2010). Vegetable fermented foods such as table olives, pickles, sauerkraut, and kimchi, are slowly overtaking the role of fermented products of animal origin (dairies and sausages) as leading source of live bacteria in human diets, and they are also increasingly considered as novel reservoirs of yet uncharacterized probiotic strains (Ranadheera et al., 2010). Table olives could therefore represent a natural source for the isolation of novel probiotic bacterial strains. This is especially true for those olives fermented with traditional procedures relying on the activity of indigenous microbial consortia of environmental origin. The microbiota of processed olives and brines includes, among others, several LAB species such as *L. plantarum, L. pentosus, L. paracasei, L. rhamnosus, Leuconostoc mesenteroides* (Arroyo-López et al., 2008; Hurtado et al., 2012; Zinno et al., 2017).

Fermented vegetable matrices are presently recognized not only as a source, but also as a vehicle of probiotic bacteria. Recent studies demonstrated that LAB species isolated from different table olive *cultivars* exhibit probiotic features, such as resistance to acid and bile salts, antimicrobial activity and interaction with intestinal epithelial cells. This suggests their potential application as novel probiotic candidates for *in vivo* studies in animals and humans (Bevilacqua et al., 2010; Abriouel et al., 2012; Argyri et al., 2013; Botta et al., 2014; Montoro et al., 2016). However, simplified *in vitro/in vivo* models represent useful and less expensive screening tools to identify probiotic strains from a large number of microbial candidates. Human intestinal epithelial Caco-2 cells are a well characterized enterocyte-like cell line, capable of expressing the morphological and functional differentiation features which are typical of mature enterocytes, including cell polarity and a functional brush border (Sambuy et al., 2005). The Caco-2 cell line has been extensively used as a reliable *in vitro* system to study the adhesion capacity of lactobacilli as well as their probiotic effects, such as protection against intestinal injury induced by pathogens (Liévin-Le Moal et al., 2002; Resta-Lenert and Barrett, 2003; Roselli et al., 2006; Montoro et al., 2016).

The nematode *Caenorhabditis elegans* is becoming an increasingly valuable *in vivo* model to study host-probiotic interactions. Its success lies in the transparency of the body, in the small size and in the absence of ethical issues. Moreover, it is inexpensive to maintain and suitable for screening studies (Clark and Hodgkin, 2014). *C. elegans* is a powerful tool to test the effects of ingested bacteria on host physiology and it can also be useful in providing mechanistic insights on the beneficial effects of probiotics. A growing number of studies employing the *C. elegans* model system demonstrated that ingestion of lactobacilli and bifidobacteria can prolong the lifespan of nematodes and modify host defense (Kim and Mylonakis, 2012; Komura et al., 2013; Park et al., 2015). The *L. gasseri* strain SBT2055, which was reported to exert beneficial effects in mice and humans, showed a positive impact on longevity and/or aging in this nematode (Nakagawa et al., 2016). The health-promoting *L. delbrueckii* subspecies *bulgaricus* was also found to increase the lifespan of nematodes (Zanni et al., 2017), further highlighting the power of this *in vivo* model.

The aim of the present study was the identification and characterization of novel potentially probiotic strains of *L. pentosus* and *L. coryniformis*, deriving from a LAB collection of isolates from Nocellara del Belice table olives (Zinno et al., 2017). We used a combination of *in vitro* and *in vivo* approaches, including Caco-2 cell cultures and the *C. elegans* nematode model, to select specific strains displaying beneficial host-microbe interactions. The autochtonous nature of the food fermenting microbiota of origin, as well as the GRAS status of LAB species, allows to employ these strains as starter cultures in food fermentations, with the added value of providing health promoting traits.

## Materials and methods

### Bacterial strains and growth conditions

The LAB strains described in this work, as well as reference strains *L. plantarum* ATCC® 14917™, *L. pentosus* ATCC® 8041™ and *L. rhamnosus* GG ATCC® 53103™ (LGG), were grown in De Man Rogosa Sharpe (MRS) medium for 24–48 h at 30 or 37°C under anaerobic conditions. The enterotoxigenic *Escherichia coli* strain K88 (ETEC, O149:K88ac, provided by The Lombardy and Emilia Romagna Experimental Zootechnic Institute, Reggio Emilia, Italy) and *E. coli* strain OP50 were grown in Luria-Bertani (LB) broth at 37°C overnight. *Salmonella enterica* serovar Typhimurium LT2 and *Listeria monocytogenes* OH were routinely grown in Tryptone Soya Broth (TSB) at 37 and 30°C, respectively. All media and supplements were provided by Oxoid (Milan, Italy).

### Species identification

For taxonomical identification, 16S rDNA gene fragments were amplified from LAB isolates using the P0-P6 primer pair (P0: 5′-GAGAGTTTGATCCTGGCT-3′; P6: 5′-CTACGGCTACCTTGTTAC-3′; Di Cello and Fani, 1996). Two μl of DNA extracted by microlysis were used for PCR amplification with the following program: 95°C for 10 min, 30 cycles at: 94°C for 1 min, 55°C for 90 s, 72°C for 150 s; one step at 55°C for 10 min followed by a final step at 72°C for 10 min. Amplified PCR fragments were analyzed by gel electrophoresis in 0.8% agarose in 1X TAE and then purified with NucleoSpin Gel and PCR clean-up purification kit (Macherey-Nagel, Germany). Sequencing of purified 16S rDNA fragments was performed at Bio-Fab Research (Italy) laboratories. For taxonomical identification, DNA sequences were compared with those reported in the BLAST NCBI (National Center for Biotechnology Information, Bethesda, USA) database. Nucleotide sequences of the amplified 16S rDNA from each LAB isolate were submitted to GenBank, and the corresponding accession numbers are reported in Table 1.

**Table 1:** List of LAB isolates deriving from Nocellara del Belice table olives fermented with Sivigliano or Castelvetrano methods and related species identified by 16S rDNA sequencing.

**Strain ID**	**Bacterial species**	**% Identity with reference species in BLAST database**	**Source of isolation**	**GenBank accession number**
C303.8	*Enterococcus gallinarum*	99	Castelvetrano	MG585222
C301.1	*Enterococcus gallinarum*	99	Castelvetrano	MG585223
C302.1	*Enterococcus casseliflavus*	99	Castelvetrano	MG585224
C302.4	*Enterococcus casseliflavus*	99	Castelvetrano	MG585225
C303.6	*Enterococcus casseliflavus*	99	Castelvetrano	MG585226
C304.2	*Leuconostoc mesenteroides*	99	Castelvetrano	MG585227
I307.27	*Leuconostoc mesenteroides*	99	Sivigliano	MG953414
G307.7	*Leuconostoc mesenteroides*	99	Sivigliano	MG585228
G3010.28	*Leuconostoc mesenteroides*	99	Sivigliano	MG585229
G3010.29	*Leuconostoc mesenteroides*	99	Sivigliano	MG585230
H306.1	*Leuconostoc mesenteroides*	99	Sivigliano	MG585231
I307.20	*Leuconostoc mesenteroides*	99	Sivigliano	MG585232
I307.22	*Leuconostoc mesenteroides*	99	Sivigliano	MG585233
I307.29	*Leuconostoc mesenteroides*	99	Sivigliano	MG585234
I306.9	*Leuconostoc mesenteroides*	99	Sivigliano	MG585235
I3010.34	*Leuconostoc mesenteroides*	99	Sivigliano	MG585236
L309.4	*Leuconostoc mesenteroides*	98	Sivigliano	MG585237
C305.2	*Leuconostoc mesenteroides*	99	Castelvetrano	MG585238
C305.16	*Leuconostoc mesenteroides*	99	Castelvetrano	MG585257
C305.5	*Lactobacillus pentosus*	99	Castelvetrano	MG585239
D301.4	*Lactobacillus pentosus*	99	Castelvetrano	MG585240
D302.23	*Lactobacillus pentosus*	98	Castelvetrano	MG585241
D302.29	*Lactobacillus pentosus*	99	Castelvetrano	MG585242
G306.1	*Lactobacillus pentosus*	99	Sivigliano	MG585243
G306.2	*Lactobacillus pentosus*	99	Sivigliano	MG585244
G308.65	*Lactobacillus pentosus*	99	Sivigliano	MG953413
H3010.5	*Lactobacillus pentosus*	99	Sivigliano	MG585245
I306.2	*Lactobacillus pentosus*	99	Sivigliano	MG585246
H308.2	*Lactobacillus pentosus*	99	Sivigliano	MG585247
I308.32	*Lactobacillus pentosus*	100	Sivigliano	MG585248
D303.36	*Lactobacillus pentosus*	99	Castelvetrano	MG585249
H3010.1	*Lactobacillus pentosus*	99	Sivigliano	MG585250
I306.12	*Lactobacillus coryniformis*	99	Sivigliano	MG585251
H307.1	*Lactobacillus coryniformis*	99	Sivigliano	MG585252
C305.1	*Lactobacillus coryniformis*	99	Castelvetrano	MG585253
H307.6	*Lactobacillus coryniformis*	99	Sivigliano	MG585254
C303.1	*Lactobacillus oligofermentans*	99	Castelvetrano	MG585255
G3010.31	*Lactobacillus oligofermentans*	99	Sivigliano	MG585256
C371.10	*Enterococcus gallinarum*	97	Castelvetrano	MG585258
C373.1	*Enterococcus gallinarum*	98	Castelvetrano	MG585259
D371.5	*Lactobacillus pentosus*	99	Castelvetrano	MG585260
D372.20	*Lactobacillus pentosus*	99	Castelvetrano	MG585261
D373.37	*Lactobacillus pentosus*	99	Castelvetrano	MG585262
I379.8	*Lactobacillus pentosus*	99	Sivigliano	MG585263
G377.8	*Lactobacillus pentosus*	99	Sivigliano	MG585264
G378.30	*Lactobacillus pentosus*	99	Sivigliano	MG585265
H376.2	*Lactobacillus coryniformis*	98	Sivigliano	MG585266
H376.5	*Lactobacillus coryniformis*	99	Sivigliano	MG585267
H377.3	*Lactobacillus coryniformis*	99	Sivigliano	MG585268

### Multiplex PCR assay

*L. plantarum*/*L. pentosus* strains were subjected to a multiplex PCR assay using the *recA* gene-based primers paraF (5′-GTC ACA GGC ATT ACG AAA AC-3′), pentF (5′-CAG TGG CGC GGT TGA TAT C-3′), planF (5′-CCG TTT ATG CGG AAC ACC TA-3′), and pREV (5′-TCG GGA TTA CCA AAC ATC AC-3′; Torriani et al., 2001). The PCR mixture included 1.5 mM MgCl_2_, the primers paraF, pentF, and pREV (0.25 μM each), 0.12 μM primer planF, 0.2 mM dNTPs, 3 U *Taq* DNA Polymerase (Invitrogen, Carlsbad, USA). Two μl of DNA extracted by microlysis were used for the reaction. PCR programs consisted of an initial denaturation step at 94°C for 3 min, followed by 30 cycles of amplification (denaturation at 94°C for 30 s, annealing at 56°C for 10 s, and elongation at 72°C for 30 s), and a final extension step at 72°C for 5 min. The PCR products were visualized on a 2% agarose gel in 1X TAE buffer and digitally captured by using ImageQuant LAS 4000 (GE Healthcare Life Sciences, Little Chalfont, UK).

### Rep-PCR fingerprinting

Two μl of DNA extracted by microlysis from selected *L. coryniformis* and *L. pentosus* strains (Microzone, Haywards Heath, UK) were used for PCR amplification with primer GTG_5_ (5′-GTGGTGGTGGTGGTG-3′), as previously described (Zinno et al., 2017), or with the ERIC primer (ERIC1R: 5′-ATGTAAGCTCCTGGGGATTCAC-3′; ERIC2: 5′-AAGTAAGTGACTGGGGTGAGCG-3′; de La Puente-Redondo et al., 2000).

For ERIC Rep-PCR, DNA amplification was carried out in 25 μl PCR mixture containing 1X PCR buffer, 1.5 mM MgCl_2_, 0.2 mM dNTPs, 3 U of *Taq* DNA Polymerase (Invitrogen, Carlsbad, USA) and 0.6 μM primers. Each cycle consisted of an initial denaturation step at 95°C for 3 min followed by 30 cycles of amplification (94°C for 1 min, 40°C for 1 min, 72°C for 1 min), and a final extension step at 72°C for 8 min. Amplified products were analyzed by gel electrophoresis (80 V for 4 h) in 1.8% agarose in 1X TAE. The gels were visualized under UV and digitally captured by using ImageQuant LAS 4000 (GE Healthcare Life Sciences, Little Chalfont, UK).

### Acid and bile salt tolerance assay

Tolerance to gastrointestinal conditions of selected *L. coryniformis* and *L. pentosus* strains was evaluated according to (Vizoso Pinto et al., 2006). Three ml of overnight bacterial suspensions were centrifuged at 5,000 × g for 15 min at 4°C and the corresponding pellet was diluted 1:1 in a sterile electrolyte solution simulating salivary juice (Simulated Salivary Juice, SSJ), composed of 6.2 g/l NaCl, 2.2 g/l KCl, 0.22 g/l CaCl_2_, 1.2 g/l NaHCO_3_ pH 6.9 in which lysozyme was added to a final concentration of 100 mg/l. The mixed suspension was incubated for 5 min at 37°C. Subsequently, the sample was diluted 3:5 with Simulated Gastric Juice (SGJ) containing 6.2 g/l NaCl, 2.2 g/l KCl, 0.22 g/l CaCl_2_, 1.2 g/l NaHCO_3_ pH 2.5 and 3 g/l pepsin, and incubated for 1 h at 37°C. After incubation, 1 ml aliquot of the sample was serially diluted and plated, in triplicate, onto MRS agar. The remaining sample was diluted 1:4 in Simulated Pancreatic Juice (SPJ) consisting of 6.4 g/l NaHCO_3_, 0.239 g/l KCl, 1.28 g/l NaCl, 0.5% bile extract, 0.1% pancreatin at pH 7.2, and incubated for 3 h at 37°C. At 2 and 3 h incubation times, 1 ml aliquots were withdrawn, serially diluted and plated on MRS agar. Aliquots of overnight inocula were also tested to determine the CFU/ml at the initial time point (t_0_) for each strain. In parallel, control samples were treated with Phosphate Buffered Saline (PBS) and subjected to the same procedure. Survival capacity was calculated as the percentage of 1– [(log CFU/ml_t0_-log CFU/ml_SPJ3h_)/log CFU/ml_t0_], where CFU/ml_SPJ3h_ represented the total viable counts (CFU/ml) for each strain at the final time point of incubation in SPJ, and CFU/ml_t0_ represented the total viable counts at the initial time point. All enzymes and salts used in the assay were provided by Sigma Aldrich (Milan, Italy).

### Antibiotic susceptibility tests

Antibiotic susceptibility was performed for a selected panel of antibiotics, namely ampicillin, tetracycline, chloramphenicol, and erythromycin, chosen as representatives of the most commonly used pharmacological classes of antimicrobials. For *L. pentosus*, each antibiotic was used at the breakpoint concentration defined by the FEEDAP Panel (EFSA, 2012). For *L. coryniformis*, which was not listed in the EFSA guidance document, we referred to the antibiotic concentrations reported by (Lara-Villoslada et al., 2007). Two μl of overnight bacterial cultures (OD_600_ = 1.3) were spotted onto MRS agar plates containing ampicillin, tetracycline, chloramphenicol or erythromycin, which were used at the following breakpoint concentrations: 2 mg/l, 32 mg/l, 8 mg/l, 1 mg/l, respectively, for *L. pentosus*, and 10 mg/l, 30 mg/l, 30 mg/l, 15 mg/l, respectively, for *L. coryniformis*. Plates were incubated for 24 h at 37°C in anaerobic conditions. Strains able to grow were considered resistant (R). The Minimum Inhibitory Concentration (MIC) for ampicillin and erythromycin of selected resistant strains was determined by broth microdilution assay, as described in (Devirgiliis et al., 2008). The antibiotic concentrations tested ranged from 1 to 20 mg/l and from 0.5 to 10 mg/l for ampicillin and erythromycin, respectively. Antibiotics were provided by Sigma Aldrich (Milan, Italy).

### Antimicrobial activity

To evaluate the antagonistic activity of *L. coryniformis* and *L. pentosus* strains against pathogens the agar double-layer diffusion method was performed (Damaceno et al., 2017). The indicator strains used were: *S. enterica* serovar Typhimurium LT2, *L. monocytogenes* OH and ETEC K88. Two μl/each of *L. coryniformis* and *L. pentosus* overnight cultures (OD_600_ = 1.3) were spotted onto MRS agar and incubated at 37°C for 24 h in anaerobic conditions. After incubation, cells were killed by chloroform exposure for 30 min. Plates were then overlaid with 7 ml TSA soft agar, which had been previously inoculated with 1% (v/v) of each pathogen indicator strain, and incubated at the corresponding optimal growth temperature for 24 h. The antagonist activity was recorded as the diameter (mm) of growth inhibition halo around each spot.

### Caco-2 cell culture and growth conditions

The human intestinal Caco-2/TC7 cell line was provided by Monique Rousset (Institute National de la Santé et de la Recherche Médicale, INSERM, France). These cells derive from parental Caco-2 cells, exhibit a more homogeneous expression of differentiation traits, and have been reported to express higher metabolic and transport activities than the original cell line, more closely resembling small intestinal enterocytes (Caro et al., 1995). The Caco-2/TC7 cells were routinely maintained at 37°C in an atmosphere of 10% CO_2_/95% air at 90% relative humidity and grown on plastic tissue culture flasks (Becton Dickinson, Milan, Italy) in Dulbecco's modified Minimum Essential Medium (complete DMEM: 3.7 g/L NaHCO_3_, 4 mM glutamine, 10% heat inactivated fetal calf serum, 1% nonessential amino acids, 10^5^ U/l penicillin and 100 mg/l streptomycin). All cell culture reagents were from Euroclone (Milan, Italy).

### Competition assay for pathogen adhesion to Caco-2 cells

Caco-2 cells were seeded in 12-well plates (Becton Dickinson) and, after confluency, were left for 14-17 days to allow differentiation (Sambuy et al., 2005). Medium was changed three times a week. Complete DMEM was replaced with antibiotic- and serum-free DMEM 16 h before the assay. To test the capacity of the selected *L. pentosus* and *L. coryniformis* strains to compete with pathogens for adhesion to Caco-2 cells, *S. enterica* serovar Typhimurium LT2 and *L. monocytogenes* OH pathogens were used as test strains. Preliminary experiments were performed to set up the optimal growth conditions for pathogens as well as for lactobacilli, to ensure that each strain could be used at the exponential growth phase. The viability of pathogens and lactobacilli in DMEM was also previously verified. On the day of the assay, overnight bacterial cultures were diluted 1:10 in LB (pathogens) or MRS media (lactobacilli) and grown for 4, 5, or 6 h to the exponential growth phase, according to the respective optimal conditions previously identified for each strain. After monitoring the OD_600_, appropriate amounts of bacterial cells were harvested by centrifugation at 5,000 × g for 10 min, resuspended in antibiotic- and serum-free DMEM and added to cell monolayers at a concentration of 1 × 10^7^ CFU/well (pathogens) or 1 × 10^8^ CFU/well (lactobacilli). Cells were incubated at 37°C for 1.5 h with either one of the two pathogens, alone or in combination with one of the two *Lactobacillus* strains. After incubation at 37°C for 1.5 h, non-adhering bacteria were removed by 5 washes with Hanks' Balanced Salt solution (HBSS: 137 mM NaCl, 5.36 mM KCl, 1.67 mM CaCl_2_, 1 mM MgCl_2_, 1.03 mM MgSO_4_, 0.44 mM KH_2_PO_4_, 0.34 mM Na_2_HPO_4_, 5.6 mM glucose) and cell monolayers were lysed with 1% Triton-X-100, according to (Roselli et al., 2006). Adhering, viable pathogen cells were quantified by plating appropriate serial dilutions of Caco-2 lysates on Violet Red Bile Glucose Agar (VRBGA, for *Salmonella*) or Listeria Selective Agar Base (Oxford, for *Listeria*). These two media were selective for pathogens, preventing the growth of lactobacilli.

### *C. elegans* strain and growth conditions

The wild-type *C. elegans* strain, Bristol N2, was grown at 16°C on Nematode Growth Medium NGM plates covered by a layer of *E. coli* OP50, LGG, *L. coryniformis*, or *L. pentosus* strains, which were prepared as described in (Zanni et al., 2015).

### *C. elegans* lifespan assay

Synchronized N2 adults were placed to lay embryos for 2 h on NGM plates, lawned with different bacteria, and then sacrificed. All lifespan assays started when the progeny became fertile (t0). Animals were transferred to new plates seeded with fresh lawns and monitored daily. They were scored as dead when they no longer responded to gentle touch with a platinum wire. At least 60 nematodes per condition were used in each experiment.

### Estimation of bacterial titer within the nematode gut

For each experiment, 10 days old animals were washed and lysed according to (Uccelletti et al., 2010). Worm lysates were then plated onto MRS-agar plates. The number of CFU was counted after 48 h of incubation at 37°C, anaerobically.

### Nematode brood size measurements

Progeny production was evaluated according to (Zanni et al., 2015) with some modifications. Briefly, synchronized worms obtained as above were grown on NGM plates seeded with bacteria and then allowed to lay embryos at 16°C. Next, animals were transferred onto a fresh bacteria plate every day, and the number of progeny was counted with a Zeiss Axiovert 25 microscope. The procedure was repeated until the mother worms stopped laying eggs. Each day the progeny production was recorded and was compared with the OP50- or LGG-fed nematodes.

### Pharyngeal pumping assay

Pharyngeal pumping was analyzed as described in (Uccelletti et al., 2008) under Zeiss Axiovert 25 microscope by counting the number of grinder contractions of 10 animals for each treatment, during a period of 30 s. The analysis was performed in 13-days-adult worms, grown on different bacteria starting from embryo stage.

### Body bending evaluation

The locomotion behavior of nematodes, fed with different bacteria from embryos hatching, was analyzed by body bending counting after 30 s. After several washes in M9 buffer to remove bacteria, nematodes were placed in 10 μl of M9 buffer allowing them to swim freely. About 10 worms for each experimental condition were monitored.

### Lipofuscin analysis

The autofluorescence of intestinal lipofuscin was measured as an index of senescence at day 13 of adulthood. Randomly selected worms from the plate lawned with bacteria were washed three times with M9 buffer. Worms were then placed onto a 3% agar pad containing 20 mM sodium azide. Lipofuscin autofluorescence was detected by fluorescence microscopy (Zeiss Axiovert 25).

### *C. elegans* killing assay

For killing assay 35 mm-NGM plates were spread with 60 μl of *L. pentosus* D303.36 or *L. coryniformis* H307.6 mixed with *S. enterica* serovar thyphimurium LT2 or *L. monocytogenes* OH, in 1:1 ratio; the strains were grown as indicated above. *C. elegans* synchronous L4 larvae were transferred onto the bacterial lawn and incubated at 25°C. Worms were monitored every day. Nematodes fed with pathogen alone were taken as control. A worm was considered dead when it failed to respond to touch.

### Statistical analysis

All experiments were performed at least in triplicate. Data are presented as mean ± SD. Prior to the analysis, normal distribution and homogeneity of variance of all variables were assumed with Shapiro-Wilk and Levene's tests, respectively. For *in vivo* experiments in *C. elegans* the statistical significance was determined by Student's *t*-test or one-way ANOVA analysis coupled with a Bonferroni post test (GraphPad Prism 5.0 software, GraphPad Software Inc., La Jolla, CA, USA). For *in vitro* experiments, statistical significance was evaluated by one-way ANOVA, followed by *post-hoc* Tukey honestly significant difference (HSD) test. Statistical univariate analysis was performed with the “Statistica” software package (version 5.0; Stat Soft Inc., Tulsa, OK). Differences with *p*-values < 0.05 were considered significant and were indicated as follows: ^*^p < 0.05, ^**^p < 0.01, and ^***^p < 0.001.

## Results

### Species identification of LAB isolates

**Figure 1:**
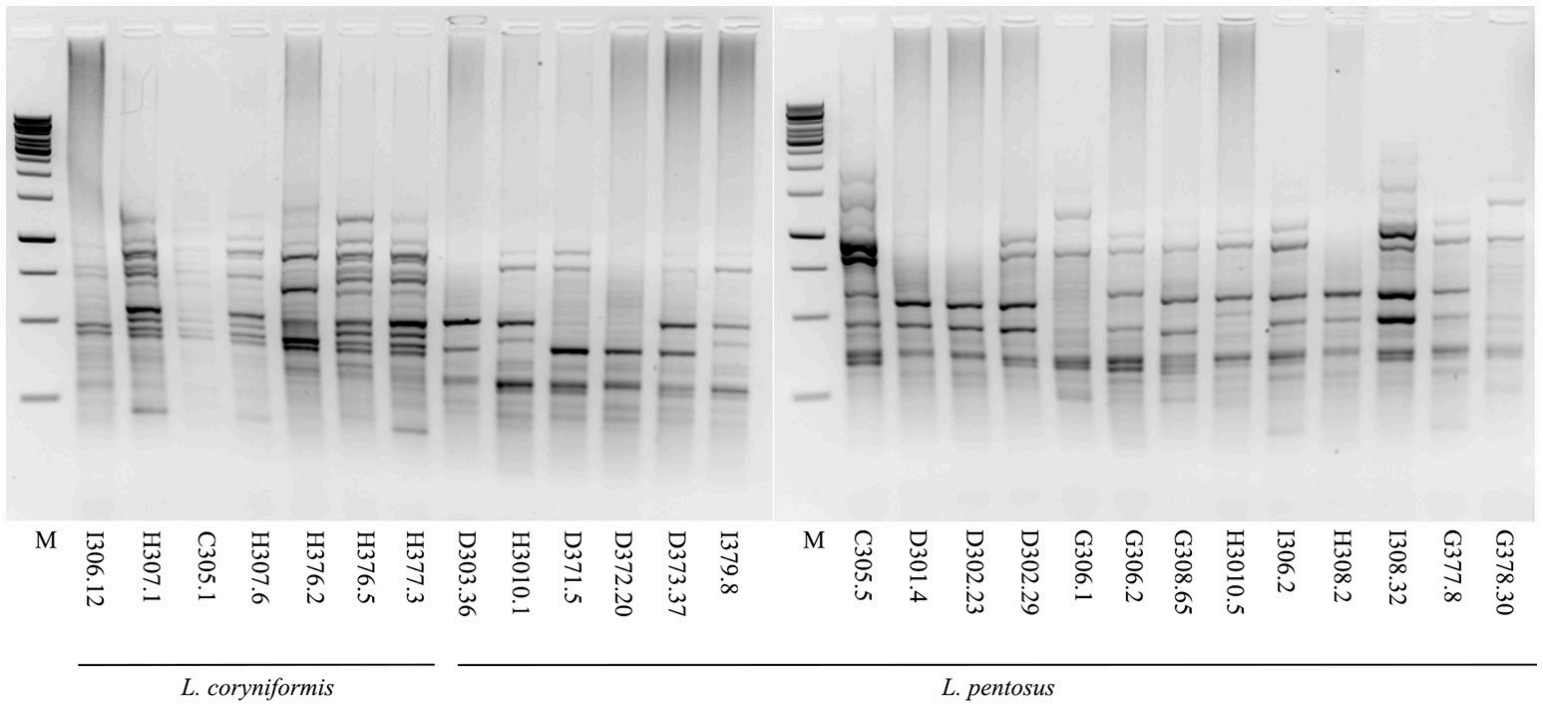
Strain typing of the selected *L. pentosus* and *L. coryniformis* isolates by rep-PCR. Agarose gel electrophoresis of GTG_5_ rep-PCR fingerprinting profiles of *L. coryniformis* and *L. pentosus* strains. M: 1 kb DNA ladder (Microzone, UK).

A total of 49 Lactic Acid Bacteria strains deriving from Nocellara del Belice table olives fermented with Spanish or Castelvetrano methods, recently collected in our previous work (Zinno et al., 2017), were characterized at the species level. Ribosomal 16S DNA sequencing allowed the identification of 4 *Enterococcus gallinarum*, 3 *E. casseliflavus*, 14 *L. mesenteroides*, 7 *L. coryniformis* and 2 *L. oligofermentans* (Table 1). For 19 isolates, however, similarity searches using sequenced ribosomal DNA fragments retrieved ambiguous species assignments, as the genomic sequences matched both *L. pentosus* and *L. plantarum* with similar scores. These isolates were therefore subjected to a multiplex PCR assay, using *recA* gene-based primers, which are able to discriminate *L. plantarum, L. paraplantarum*, and *L. pentosus* species (Torriani et al., 2001). The PCR results clarified that all 19 strains belonged to the *L. pentosus* species (Figure S1). *L. pentosus* and *L. coryniformis* isolates were chosen as potential probiotic candidates for further screening, as these two species are widely employed as starter and/or protective cultures in table olive manufacturing. To assess the presence of unique strains, these isolates were subjected to strain typing using a combination of GTG_5_ rep-PCR (Figure 1) and of Enterobacterial Repetitive Intergenic Consensus sequence PCR (ERIC-PCR, data not shown). The results shown in Figure 1 revealed the presence of distinct fingerprinting profiles characterizing each strain within both species, indicating that each of the 19 *L. pentosus* and 7 *L. coryniformis* isolates represented a unique strain. Therefore, they were all subjected in parallel to the subsequent assays aimed at characterizing probiotic capacity.

### *In vitro* tolerance to simulated gastro-intestinal conditions

Tolerance to gastrointestinal conditions was assayed using a series of sequential treatments that simulate bacterial transit along the mammalian GI tract. As shown in Table 2, each treatment differentially affected survival of the tested strains. In particular, 1 h incubation in SGJ, characterized by acidic pH (2.5), exerted a mild but significant reduction of bacterial counts for all the *L. coryniformis* and *L. pentosus* strains. Percent survival was close to 90% in all cases, with the exception of *L. coryniformis* isolate I306.12 which was almost unaffected by this treatment (98% survival), while two other *L. coryniformis* strains (C305.1 and H376.2) were unable to survive this condition (Table 2). Subsequent treatment of the surviving strains with SPJ containing bile salts and pancreatin, displayed a more severe impact on overall bacterial survival, as revealed by the inability of 5 *L. pentosus* strains to tolerate pancreatic conditions after 2 h of incubation (Table 2). Both 2 and 3 h treatments in SPJ are usually employed to mimick transit time in the GI tract. In our case however, 3 h incubation in SPJ did not affect overall bacterial survival any further than the 2 h incubation timepoint, with the exception of the *L. pentosus* strain G378.30 which did not survive, and of 5 *L. pentosus* strains that showed a decrease of about 1.5 log in CFU/ml. In order to compare the results obtained, survival capacity was calculated for each strain as well as for the reference probiotic strain LGG (see Materials and Methods). Overall, the capacity of the tested strains to tolerate gastrointestinal conditions ranged between 50 and 80%. Notably, 7 *L. pentosus* and 4 *L. coryniformis* strains showed survival capacities similar or higher than those of the reference probiotic strain LGG, namely about 70% at the end of both treatments (Table 2).

**Table 2:** Survival of *L. pentosus* and *L. coryniformis* strains under *in vitro* simulated gastro-intestinal conditions.

**Bacterial species**	**Strain ID**	**t0**	**SGJ**	**SPJ _2h_**	**SPJ _3h_**	**Survival capacity (%)**
*L. pentosus*	C305.5	8.20 ± 0.02^a^	7.53 ± 0.03^b^	6.18 ± 0.02^c^	6.14 ± 0.03^c^	74.92
	D301.4	9.12 ± 0.08^a^	7.75 ± 0.15^b^	0	0	0
	D302.23	8.93 ± 0.03^a^	8.40 ± 0.07^b^	0	0	0
	D302.29	9.23 ± 0.003^a^	8.70 ± 0.05^b^	0	0	0
	G306.1	8.31 ± 0.18^a^	7.77 ± 0.05^b^	5.97 ± 0.07^c^	5.94 ± 0.07^c^	71.44
	G306.2	8.91 ± 0.12^a^	8.43 ± 0.02^b^	6.99 ± 0.02^c^	6.91 ± 0.05^c^	77.60
	G308.65	9.09 ± 0.06^a^	8.30 ± 0.02^b^	0	0	0
	H3010.5	9.49 ± 0.09^a^	8.48 ± 0.05^b^	6.20 ± 0.12^c^	4.72 ± 0.15^d^	49.76
	I306.2	8.61 ± 0.13^a^	8.07 ± 0.09^b^	6.69 ± 0.03^c^	6.50 ± 0.10^c^	75.47
	H308.2	8.63 ± 0.24^a^	8.10 ± 0.05^b^	6.66 ± 0.04^c^	5.99 ± 0.10^d^	69.43
	I308.32	9.19 ± 0.01^a^	8.06 ± 0.02^b^	0	0	0
	G377.8	9.38 ± 0.32^a^	8.23 ± 0.01^b^	6.77 ± 0.02^c^	6.44 ± 0.07^c^	68.64
	G378.30	9.17 ± 0.19^a^	8.08 ± 0.07^b^	5.52 ± 0.30^c^	0	0
	D303.36	9.05 ± 0.14^a^	8.54 ± 0.03^b^	5.21 ± 0.07^c^	4.68 ± 0.04^d^	51.65
	H3010.1	8.92 ± 0.21^a^	6.81 ± 0.35^b^	5.01 ± 0.06^c^	4.74 ± 0.15^c^	53.17
	D371.5	8.66 ± 0.04^a^	7.84 ± 0.04^b^	5.62 ± 0.51^c^	5.32 ± 0.05^c^	61.43
	D372.20	9.10 ± 0.06^a^	7.85 ± 0.03^b^	5.99 ± 0.03^c^	4.56 ± 0.33^d^	50.09
	D373.37	8.53 ± 0.05^a^	7.57 ± 0.09^b^	6.27 ± 0.2^c^	6.23 ± 0.02^c^	72.99
	I379.8	8.95 ± 0.04^a^	8.25 ± 0.03^b^	7.02 ± 0.04^c^	6.88 ± 0.02^d^	76.88
*L. coryniformis*	I306.12	8.71 ± 0.093^a^	8.56 ± 0.12^a^	6.80 ± 0.25^b^	6.71 ± 0.03^b^	77.04
	H307.1	8.80 ± 0.09^a^	8.09 ± 0.13^b^	6.13 ± 0.06^c^	6.18 ± 0.04^c^	70.20
	C305.1	8.25 ± 0.01	0	0	0	0
	H307.6	9.66 ± 0.24^a^	8.54 ± 0.04^b^	7.80 ± 0.05^c^	7.09 ± 0.10^d^	73.44
	H376.2	8.55 ± 0.05	0	0	0	0
	H376.5	8.35 ± 0.01^a^	7.54 ± 0.14^b^	5.55 ± 0.41^c^	5.49 ± 0.15^c^	65.82
	H377.3	8.76 ± 0.06^a^	8.21 ± 0.04^b^	6.98 ± 0.04^c^	6.96 ± 0.01^c^	79.49
*L. rhamnosus*	GG	8.7 ± 0.021^a^	8.1 ± 0.39^b^	6.9 ± 0.04^c^	6.1 ± 0.04^d^	69.91

*Values represent mean log (CFU/ml) ± standard deviation. Distinct letters indicate significance at p value < 0.05 among log (CFU/ml) values for the different tested conditions, within each strain*.

*Survival capacity is expressed as the percentage of 1– [(log CFU/ml_t0_ – log CFU/ml_SPJ3h_)/log CFU/ml_t0_]*.

### Antibiotic resistance

The *L. coryniformis* and *L. pentosus* strains were analyzed for resistance to ampicillin, tetracycline, chloramphenicol, and erythromycin, as representatives of different pharmacological classes of antimicrobials widely employed in human and veterinary medicine. The selected antibiotics were used at the breakpoint concentrations proposed by the European Food Safety Agency (EFSA) for *L. pentosus* (EFSA, 2012), while for *L. coryniformis* they were chosen according to (Lara-Villoslada et al., 2007). Table 3 shows the antibiotic resistance panel for each tested bacterial strain. Overall, the majority of the tested strains displayed susceptibility to the selected antibiotics. However, all *L. pentosus* strains displayed resistance to ampicillin, with the exception of isolate C305.5. As for the other antibiotics, all 19 *L. pentosus* strains were sensitive to tetracycline and chloramphenicol, while 3 strains, namely D303.36, H3010.1, and I379.8, displayed phenotypic resistance to erythromycin. All *L. coryniformis* strains resulted susceptible to all tested antibiotics (Table 3). The ampicillin and erythromycin resistant *L. pentosus* strains were further investigated by quantifying their MIC values for the two antibiotics. MIC values above the breakpoints, corresponding to 2 mg/l for ampicillin and 1 mg/l for erythromycin, were considered indicative of phenotypic antibiotic resistance. The results show that ampicillin MIC values ranged between 2 and 20 mg/l, with variable distribution within the 18 analyzed strains. Three of them, namely *L. pentosus* D303.36, H3010.1, and I379.8, which were resistant to both antibiotics, also displayed erythromycin MIC values of 1.125 or 2.5 mg/l (Table 3).

**Table 3:** Antibiotic resistance of *L. pentosus* and *L. coryniformis* strains.

		**Antibiotic*^a^***			
**Bacterial species**	**Strain ID**	**Ampicillin (2 mg/l)**	**Tetracycline (32 mg/l)**	**Chloramphenicol (8 mg/l)**	**Erythromycin (1 mg/l)**
*L. pentosus*	C305.5	S	S	S	S
	D301.4	R (20)	S	S	S
	D302.23	R (20)	S	S	S
	D302.29	R (2)	S	S	S
	G306.1	R (8)	S	S	S
	G306.2	R (4)	S	S	S
	G308.65	R (16)	S	S	S
	H3010.5	R (4)	S	S	S
	I306.2	R (8)	S	S	S
	H308.2	R (12)	S	S	S
	I308.32	R (8)	S	S	S
	G377.8	R (4)	S	S	S
	G378.30	R (8)	S	S	S
	D303.36	R (16)	S	S	R (2.5)
	H3010.1	R (4)	S	S	R (1.25)
	D371.5	R (4)	S	S	S
	D372.20	R (8)	S	S	S
	D373.37	R (8)	S	S	S
	I379.8	R (4)	S	S	R (2.5)
		**Ampicillin (10 mg/l)**	**Tetracycline (30 mg/l)**	**Chloramphenicol (30 mg/l)**	**Erythromycin (15 mg/l)**
*L. coryniformis*	I306.12	S	S	S	S
	H307.1	S	S	S	S
	C305.1	S	S	S	S
	H307.6	S	S	S	S
	H376.2	S	S	S	S
	H376.5	S	S	S	S
	H377.3	S	S	S	S

a *Each antibiotic was used at the microbiological breakpoint indicated in parenthesis, according to the bacterial species*.

*LAB strains resulting sensitive or resistant to antibiotic are referred as S or R, respectively*.

*MIC values (expressed as mg/l) for resistant strains are indicated in parenthesis*.

### Antimicrobial activity against pathogens

The great majority of the *L. pentosus* and *L. coryniformis* strains displayed antagonistic activity in the agar double-layer diffusion test against all three pathogens chosen as indicator strains (*S. enterica* serovar Typhimurium LT2, *L. monocytogenes* OH, and ETEC K88). The strength of such inhibition was variable among the different lactobacilli, as shown by broad variability of the inhibition halo diameters on all 3 pathogen test strains (Table S1). The only exception was represented by *L. pentosus* strain C305.5, which was unable to inhibit growth of *S. enterica* serovar Typhimurium LT2 (Table 4). In particular, the majority of the 19 *L. pentosus* strains produced inhibition halo diameters above median value when tested against all three pathogens. The *L. coryniformis* strains, on the other hand, showed overall inhibition halo diameters between 1 mm and the corresponding median value against all three pathogens, with the exception of two strains, namely H307.1 and H377.3, both displaying inhibition halo diameters above the median against *Salmonella* and ETEC (Table 4). Notably, both of these latter strains had shown survival capacities comparable to that of the well characterized probiotic strain LGG following simulated gastro-intestinal condition, and were both susceptible to all tested antibiotics.

**Table 4:** Antimicrobial activity of *L. pentosus* and *L. coryniformis* strains against indicator pathogens.

		**Pathogen strain**		
**Bacterial species**	**Strain ID**	***S. enterica* serovar Typhimurium LT2**	***L. monocytogenes* OH**	**ETEC K88**
*L. pentosus*	C305.5	−	+	+
	D301.4	++	+	++
	D302.23	++	++	++
	D302.29	++	+	+
	G306.1	++	+	+
	G306.2	++	++	++
	G308.65	++	+	+
	H3010.5	+	++	++
	I306.2	++	++	++
	H308.2	++	+	++
	I308.32	++	++	+
	G377.8	+	++	++
	G378.30	+	++	++
	D303.36	++	+	+
	H3010.1	++	++	++
	D371.5	+	++	++
	D372.20	+	++	++
	D373.37	+	+	++
	I379.8	+	++	++
*L. coryniformis*	I306.12	+	+	+
	H307.1	++	+	++
	C305.1	+	+	+
	H307.6	+	+	+
	H376.2	+	+	+
	H376.5	+	+	+
	H377.3	++	+	++

Inhibitory activities refer to the measured inhibition halo diameter and are indicated as: – (diameter <1 mm);

*+ (1 mm < diameter < median value); ++ (diameter > median value)*.

### *In vivo* screening in *C. elegans*

*In vivo* screening of the *L. pentosus* and *L. coryniformis* strains for health-promoting traits was performed in the *C. elegans* model system, which was shown to display beneficial effects in response to administration of probiotic bacteria (Nakagawa et al., 2016). Lifespan analysis was initially used as a pre-screening assay to select those bacterial strains able to prolong worm longevity. To this aim, the lifespan of animals separately fed each of the isolated *Lactobacillus* strains starting from embryo hatching, was compared with that of control worms grown on LGG as the only bacterial source, or on the standard *E. coli* OP50 diet. The results are reported in Figure S2. Among the tested strains, the *L. pentosus* D303.36 diet induced a relevant increase in *C. elegans* longevity (Figure S2A), and animals fed *L. coryniformis* H307.6 showed similar viability with respect to those fed the probiotic strain LGG (Figure S2B). On the other hand, when compared to the control OP50 diet, none of the other tested strains determined significant increase in worm lifespan, with the exception of *L. pentosus* D371.5 (Figure S2A) and *L. coryniformis* H376.2 (Figure S2B), which led to significant lifespan reduction when used as the sole dietary source of bacteria. The *L. coryniformis* strain I306.12 induced an embryonic lethal phenotype, since embryos failed to develop into larvae (data not shown).

**Figure 2:**
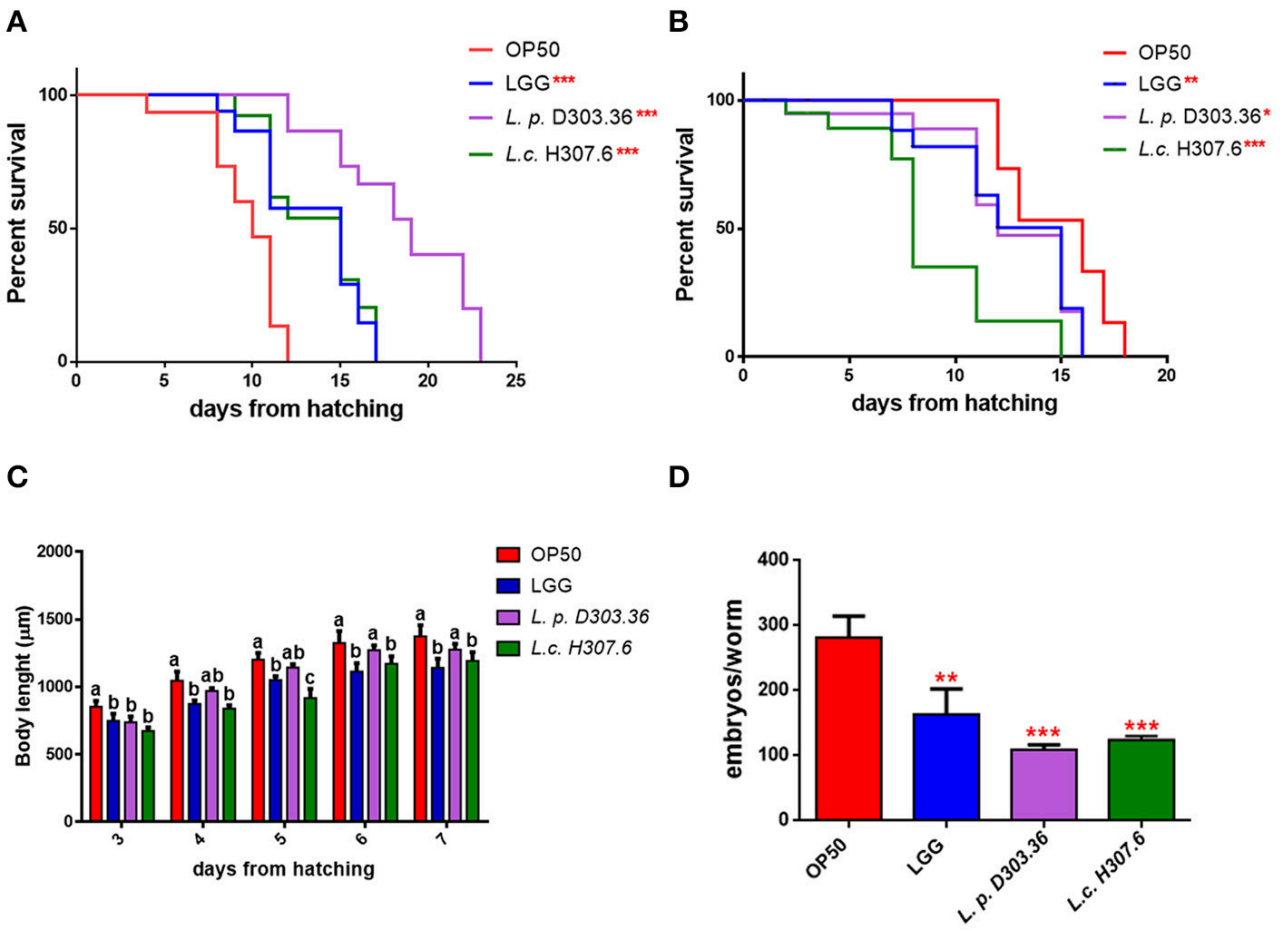
Effect of the *L. pentosus* D303.36 and *L. coryniformis* H307.6 strains on *C. elegans* lifespan, body length, and fertility. **(A)** Kaplan–Mèier survival plots of N2 fed *L. p*. D303.36 and *L. c*. H307.6 strains, starting from embryo hatching; *n* = 60 for each single experiment. Lifespans of OP50- and LGG-fed animals are reported as controls. **(B)** Survival of *C. elegans* fed heat killed bacterial strains. Statistical analysis was evaluated by one-way ANOVA with the Bonferroni *post-test*; asterisks indicate significant differences (^*^p < 0.05; ^**^p < 0.01; ^***^p < 0.001). **(C)** Effect of bacteria on larval development. Worm length was measured from head to tail at the indicated time points. Statistical analysis was performed by one-way ANOVA with the Bonferroni *post-test*; different letters indicate significant differences (*p* < 0.05). **(D)** Embryo production per worm in animals fed different bacterial strains. Bars represent the mean of three independent experiments (^**^p < 0.01; ^***^p < 0.001).

Therefore, the two *L. pentosus* D303.36 and *L. coryniformis* H307.6 strains were considered as the most promising candidates in terms of health-promoting features, and were selected for further analysis. Figure 2A shows that nematode median survival was recorded at days 18 and 15 when worms were fed *L. pentosus* D303.36 and *L. coryniformis* H307.6, respectively, as compared to 9.5 days in the case of OP50-fed worms. The above described effects on the longevity phenotype were not observed when worms were fed heat-killed bacteria, as shown in Figure 2B.

Microscopic observation allowed to evaluate other physiological effects promoted by the *Lactobacillus* strains in *C. elegans*: animals fed *L. pentosus* D303.36 or *L. coryniformis* H307.6 displayed reduced size with respect to OP50-fed animals along all developmental stages, similarly to the effect of feeding the probiotic strain LGG (Figure 2C). Moreover, *C. elegans* progeny production was significantly reduced when nematodes were fed *L. pentosus* D303.36 or *L. coryniformis* H307.6, with about 60% reduction of progeny number in both cases as compared to OP50-fed animals. A similar reduction was also observed in the case of LGG-fed animals, although to a lesser extent (Figure 2D).

**Figure 3:**
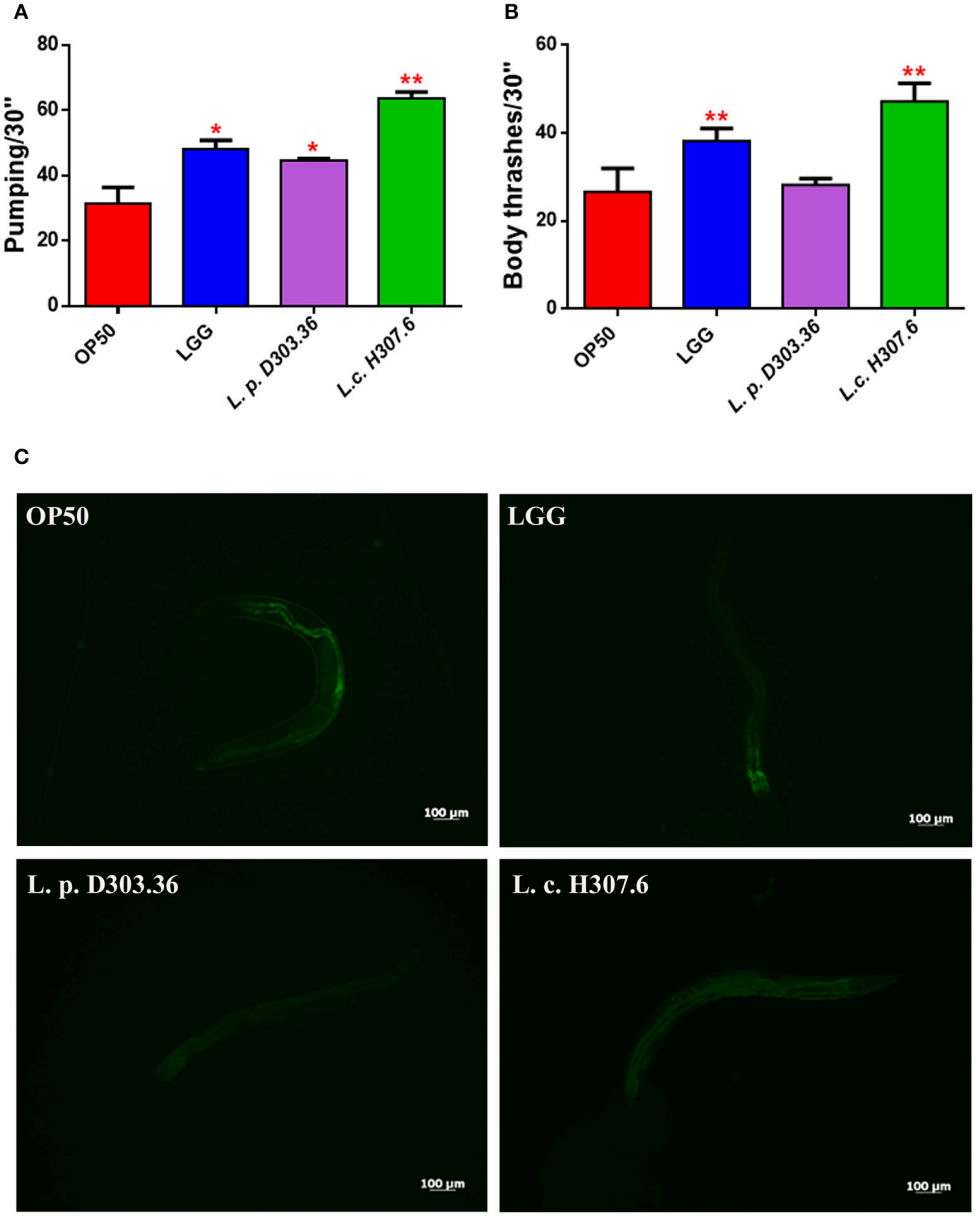
Analysis of aging markers in *C. elegans* fed the *L. pentosus* D303.36 and *L. coryniformis* H307.6 strains. **(A)** Pumping rate of 13-days-old worms, measured for 30 s and determined from the mean of 10 worms for each bacterial strain. Worms fed OP50 or LGG were used as controls. **(B)** Body bend frequency, measured for 30 s, of *C. elegans* fed different *Lactobacillus* strains or OP50. Statistical analysis was performed by one-way ANOVA with the Bonferroni *post-test*; asterisks indicate significant differences (^*^p < 0.05, ^**^p < 0.01). **(C)** Autofluorescence of lipofuscin granules in *C. elegans* fed different bacterial strains on day 13. Ten worms were used for each measurement. Scale bar = 100μm.

Subsequently, aging biomarkers were analyzed in order to evaluate the prolonged lifespan of *C. elegans* supplemented with the different isolates at 13 days of adulthood. The neuromuscular functionality of nematodes was investigated by measuring contractions of the pharynx to assess whether *L. pentosus* D303.36 and *L. coryniformis* H307.6 strains could impact on *C. elegans* swallowing capacity. Pharyngeal pumping rates increased when each of the two strains were administered to nematodes, with respect to the OP50 control (Figure 3A). In parallel, analysis of the locomotion behavior was performed to determine possible modifications of *C. elegans* mobility. Similarly to LGG-fed worms, body bending was increased when feeding *L. coryniformis* H307.6 as compared to OP50. On the other hand, the *L. pentosus* D303.36-based diet did not show any effect (Figure 3B). Evaluation of intestinal lipofuscin accumulation was performed as an additional aging biomarker. Fluorescence microscope analysis revealed a reduced fluorescent signal, diffused throughout the body of nematodes fed *L. pentosus* D303.36 or *L. coryniformis* H307.6. Similar results were obtained when feeding the probiotic control strain LGG. By contrast, nematodes fed OP50 showed intense fluorescence accumulating in large granules along the intestine, typical of aged animals (Figure 3C).

To evaluate their colonization capacity, bacteria were recovered from *C. elegans* gut and quantified by measuring CFUs at 10-days adulthood stage. Both *L. pentosus* D303.36 and *L. coryniformis* H307.6 strains showed a colonization capacity almost identical to that of the probiotic control strain LGG (data not shown).

**Figure 4:**
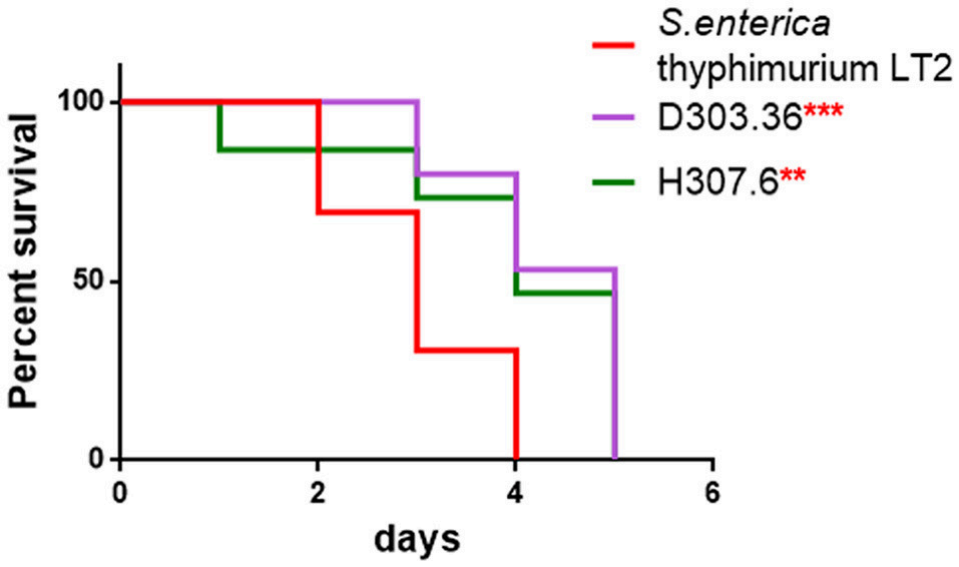
Rescuing potential of *L. pentosus* D303.36 and *L. coryniformis* H307.6 against *Salmonella enterica* infection. Kaplan–Mèier Survival plot of *C. elegans* fed *L. pentosus* D303.36 or *L. coryniformis* H307.6 in a 1:1 co-colture with *S. enterica* serovar Thyphimurium LT2. Worms fed *Salmonella* alone were taken as control (^**^p < 0.01; ^***^p < 0.001).

Probiotic strains were reported to protect *C. elegans* against infection mediated by several pathogens (Park et al., 2014; Neuhaus et al., 2017). Since both *L. pentosus* D303.36 and *L. coryniformis* H307.6 strains displayed antimicrobial activity against the three tested pathogens (Table 4), these two LAB strains were evaluated also for their protective potential in *C. elegans* against pathogen infection mediated death. *S. enterica* serovar Thyphimurium LT2 or *L. monocytogenes* OH were chosen for the assay as they represent important foodborne pathogens. The results in Figure 4 demonstrate that *C. elegans* displayed reduced survival on NGM medium when fed *S. enterica* Thyphimurium LT2 alone, as compared to nematodes supplemented with co-cultures of the same pathogen with *L. pentosus* D303.36 or *L. coryniformis* H307.6. In the case of *L. monocytogenes* OH, on the other hand, neither one of the co-cultures was able to abrogate premature death of the animals (data not shown).

### Reduction of pathogen adhesion to human intestinal epithelial cells by *L. pentosus* D303.36 and *L. coryniformis* H307.6 strains

Several pathogens, including *S. enterica* serovar Typhimurium, *L. monocytogenes* and ETEC, are able to adhere to the brush border of intestinal cells, damaging the structure of tight or adherens junctions (Boyle and Finlay, 2003; Köhler et al., 2007). Increasing evidence highlights the capacity of lactobacilli to inhibit pathogen adhesion to the intestinal mucosa and counteract the associated inflammatory processes, thus preventing intestinal disease in both humans and animals (Zhou et al., 2010; Asahara et al., 2011). The two *L. pentosus* D303.36 and *L. coryniformis* H307.6 strains were therefore analyzed for their capacity to reduce pathogen adhesion to Caco-2 cells, which represent a valuable *in vitro* model of human intestinal epithelium. Both strains, which were previously tested for their adhesion ability to Caco-2 cells (data not shown), were co-cultured with intestinal cells in combination with *S. enterica* serovar Typhimurium LT2 or *L. monocytogenes* OH.

**Figure 5:**
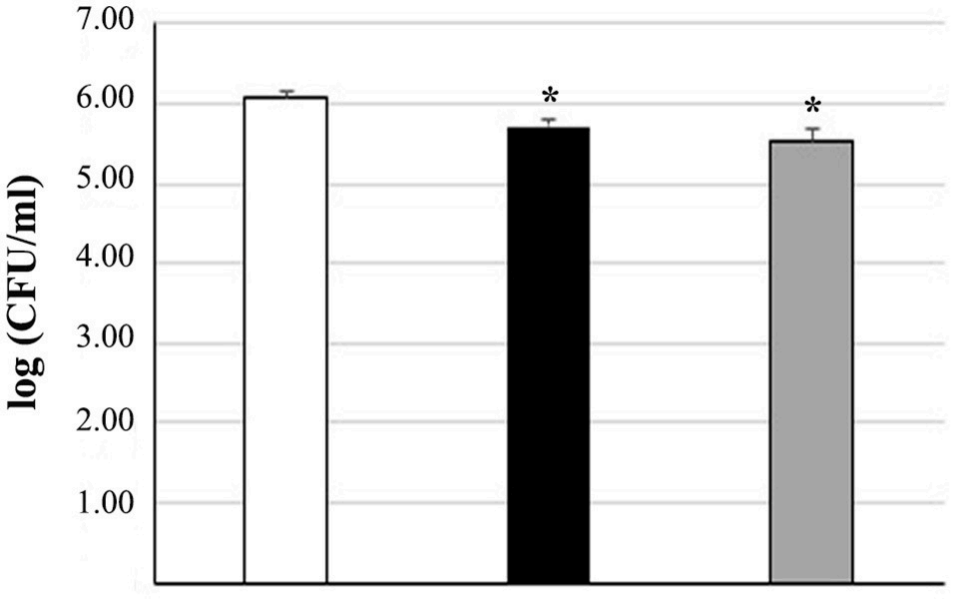
Reduction of *Salmonella enterica* adhesion to Caco-2 cells by *L. pentosus* D303.36 and *L. coryniformis* H307.6. Cell counts of viable *S. enterica* serovar Thyphimurium LT2 adhering on differentiated Caco-2 cells treated with: *S. enterica* alone (control, white column); *S. enterica* in combination with *L. pentosus* D303.36 (black column) or *L. coryniformis* H307.6 (gray column). Columns represent the mean ± SD of four independent experiments. Data are reported as log of bacterial CFU recovered after plating. Statistical analysis was performed by one-way ANOVA, followed by *post-hoc* Tukey honestly significant difference (HSD) test. Asterisks indicate significant differences (^*^p < 0.05 vs. control).

Treatment of intestinal cells with *L. pentosus* D303.36 or *L. coryniformis* H307.6 reduced adhesion of *S. enterica* serovar Typhimurium LT2 by about 0.5 log CFU/ml (Figure 5). Statistical analysis performed by ANOVA revealed *p*-values < 0.05, indicating that, although to a mild extent, both strains were able to significantly counteract pathogen attachment to the cells. On the other hand, no protective effect of *L. pentosus* D303.36 or *L. coryniformis* H307.6 against *L. monocytogenes* OH could be observed (data not shown).

## Discussion

Table olives are increasingly recognized as a potential natural source of probiotic bacteria that could be exploited to obtain a health-promoting functional product (Bonatsou et al., 2017). Different olive *cultivars* are characterized by specific autochtonous fermenting microbiota (Heperkan, 2013), representing a valuable reservoir of novel strains of environmental origin. In particular, Nocellara del Belice is an important Italian olive *cultivar*, awarded the official PDO designation (Protected Designation of Origin, EC Regulation No 134/1998), whose microbial composition is dominated by several LAB species of technological and health-related interest (Aponte et al., 2010, 2012; Zinno et al., 2017). In the present work, a combination of *in vitro* and *in vivo* approaches was used to select novel potentially probiotic *Lactobacillus* strains deriving from a LAB collection of isolates from Nocellara del Belice table olives fermented with Spanish or Castelvetrano methods, that was previously established in our laboratory (Zinno et al., 2017). Characterization of LAB isolates at the species level identified *Leuconostoc mesenteroides, L. pentosus*, and *L. coryniformis* as the predominant species, along with *L. oligofermentans, E. gallinarum*, and *E. casseliflavus* as minor components. Among these isolates, *L. pentosus* and *L. coryniformis* were chosen as potential probiotic candidates for further analysis since increasing experimental evidence reports on health-promoting features displayed by several strains belonging to these species (Olivares et al., 2006; Abriouel et al., 2017; Bendali et al., 2017). Probiotic traits are known to be strain-specific (Saulnier et al., 2009; Amund, 2016), it is therefore important to consider that all selected *L. pentosus* and *L. coryniformis* isolates analyzed in this work displayed distinct fingerprinting profiles, therefore representing unique and novel strains.

Resistance to the harsh conditions of the upper GI tract is a key pre-requisite for efficient colonization by a probiotic strain (Dicks and Botes, 2010). As a first predictive phenotypic trait, tolerance to gastrointestinal conditions was assessed by evaluating the survival capacity of each strain in comparison with the well-characterized, commercial probiotic strain *L. rhamnosus* GG. Overall, the majority of the investigated strains showed 50–80% survival capacity to gastric and pancreatic juice treatments, with strain-dependent variability. Notably, 11 of the 26 *Lactobacillus* strains assayed in this study displayed survival rates equal or higher than that of the well characterized probiotic control strain LGG (70%) at the end of simulated digestion treatments, thus pointing at the diet, and to fermented foods in particular, as a relevant source of live microorganisms that can reach the host gut microbiota in a metabolically active state, where they can transiently colonize and interact with resident gut bacteria. An important trait to be verified for safety purposes concerns antibiotic resistance profiling (Imperial and Ibana, 2016). To this aim, all the *L. coryniformis* and *L. pentosus* strains were also analyzed for resistance to ampicillin, tetracycline, chloramphenicol or erythromycin, as representatives of distinct pharmacological classes of antimicrobials commonly used in human and veterinary medicine (Aminov, 2017). While the majority of the tested strains were susceptible to these antibiotics, all but one of the *L. pentosus* isolates showed phenotypic resistance to ampicillin, with a few of them diplaying erythromycin resistance as well. These strains will be subjected to further analysis to identify the corresponding antibiotic-resistance determinants and their genomic contexts, so that possible horizontal transmission can be ascertained.

Probiotics are known to be effective in preventing or counteracting foodborne infections by reducing the growth of enteric pathogens through different mechanisms, involving competitive exclusion or the production of inhibitory molecules (Karami et al., 2017; Mathipa and Thantsha, 2017). We therefore tested the antimicrobial activity exerted by the *L. pentosus* and *L. coryniformis* strains against three common pathogens, namely *S. enterica* serovar Typhimurium, *L. monocytogenes* and ETEC. The great majority of the *Lactobacillus* isolates screened in this work resulted to be active against all three pathogens *in vitro*, although with variable, strain-specific efficacy.

At the end of this *in vitro* screening, 3 *L. coryniformis* strains appeared to be good candidate probiotics on the basis of their positive performance under simulated gastro-intestinal digestion, antibiotic susceptibility, and antimicrobial activity. However, only one of these strains was also selected by parallel screening for health-promoting traits in the *in vivo* nematode model *C. elegans*. This simplified model organism lives on bacteria as the only food source, but a substantial number of bacterial cells escape the grinding capacity of the worm larynx and can proceed to colonize the nematode gut (Nakagawa et al., 2016). The *L. coryniformis* H307.6 strain was able to significantly increase *C. elegans* lifespan as compared to the OP50 control strain, overlapping the effect exerted by the well characterized probiotic strain LGG, while positively impacting also on other well-established aging biomarkers such as pharyngeal pumping rate, body size, brood size, and lipofuscin (Lee et al., 2015). These results further confirm the ability of specific LAB strains to extend nematode lifespan as reported in previous studies (Ikeda et al., 2007; Komura et al., 2013; Nakagawa et al., 2016). Moreover, the *L. coryniformis* H307.6 displayed health-promoting activities also in host defense against *S. enterica* serovar Typhimurium, both *in vitro* (inhibiting pathogen growth as well as competing with pathogen for intestinal cell adhesion), and *in vivo* (increasing survival of infected worms). It is worth mentioning in this respect that host-pathogen interactions have been investigated in *C. elegans* for a number of pathogens of human and animal origin (Clark and Hodgkin, 2014), including *S. enterica* and *L. monocytogenes* which are able to colonize the worm gut and infect the nematode (Aballay et al., 2000; Thomsen et al., 2006). A second isolate, namely *L. pentosus* D303.36, was positively selected in *C. elegans* as a lifespan extending, health-promoting strain. This strain, however, did not display good performance with respect to tolerance to GI tract conditions, and was also resistant to ampicillin and erythromycin. Therefore, further molecular characterization is necessary to exclude potential horizontal transmission of antibiotic resistance before it can be considered a promising probiotic candidate.

On the other hand, *L. coryniformis* strains H307.1 and H377.3, that were selected as very good performers in the initial *in vitro* testing screens, were both antibiotic susceptible, as well as capable of inhibiting pathogen growth in the agar double-layer diffusion assay. However, neither one of these two strains could positively impact on *C. elegans* longevity. Table 5 summarizes the main features displayed by the candidate probiotic strains identified in this study. In light of the recent findings indicating that probiotic capacity of mixed foodborne microbial consortia might be more effective than single strain supplementation (Foligné et al., 2016; Roselli et al., 2017), testing these strains as members of a multistrain probiotic complex could open new avenues for their applications in vegetable food fermentations.

**Table 5:** Summary table listing the main features displayed by the candidate probiotic strains identified in this study.

**Species/strain ID**	***C. elegans***		**GI tract survival (%)**	**Antimicrobial activity**			**Antibiotic resistance**	
	**Longevity**	**Colonization**		***In vitro***	**Caco-2**	***C. elegans***	Growth on antibiotic^*^	**MIC (mg/ml)**
*L. coryniformis H307.1*	−	ND	70.20	+++	ND	ND	S	NA
*L. coryniformis H377.3*	−	ND	79.49	+++	ND	ND	S	NA
*L. coryniformis H307.6*	+	+	73.44	+	+(*Salmonella*)	+(*Salmonella*)	S	NA
*L. pentosus D303.36*	++	+	51.65	++	+(*Salmonella*)	+(*Salmonella*)	R(Amp, Ery)	Amp = 16Ery = 2.5

* *growth in the presence of breakpoint concentration of antibiotics; R, Resistant to the specified antibiotics; S, Susceptible to all tested antibiotics*.

*MIC, Minimum Inhibitory Concentration, determined only for strains which survived breakpoint concentrations for the specified antibiotics*.

*ND, Not Determined; NA, Not Applicable*.

## Conclusions

Extensive *in vitro* and *in vivo* characterization of 26 *Lactobacillus* strains previously isolated from fermented Nocellara del Belice table olives, led to the identification of various potential candidate probiotics. Of these, three *L. coryniformis* strains displayed good probiotic features *in vitro*, although only one of them could also exert prolongevity and protective effects in the simplified model organism *C. elegans*. The GRAS status of Lactobacilli allows to consider their application as starters of fermentation with probiotic added value. However, further validation in *in vivo* trials with more complex animal or human systems should be performed to gain deeper understanding of their potential health promoting features for human health.

## Author contributions

CD and DU conceived and designed the experiments. CD and DU wrote the paper. GP and CP critical revision of manuscript. ES performed animal experiments/treatments. PZ and BG performed microbiological analyses. MR and BG performed cell culture experiments.

### Conflict of interest statement

The authors declare that the research was conducted in the absence of any commercial or financial relationships that could be construed as a potential conflict of interest.
