# Supplementary material for: In Vitro and in Vivo Selection of Potentially Probiotic Lactobacilli From Nocellara del Belice Table Olives
Source: Front Microbiol. 2018 Mar 28;9:595. doi: 10.3389/fmicb.2018.00595 (PMC5882814; doi:10.3389/fmicb.2018.00595)
Supplement: Supplementary file 1 [file Table1.DOCX]

**Table S1. Antimicrobial activity of *L. pentosus* and *L. coryniformis* strains against indicator pathogens expressed as mm inhibition halo diameters**

|  |  | **Pathogen strain** | | |
| --- | --- | --- | --- | --- |
| **Bacterial species** | **Strain ID** | ***S. enterica* serovar Typhimurium LT2** | ***L. monocytogenes* OH** | **ETEC K88** |
| ***L. pentosus*** | C305.5 | 0 | 3 | 2 |
|  | D301.4 | 28 | 19 | 29 |
|  | D302.23 | 45 | 33 | 25 |
|  | D302.29 | 28 | 19 | 8 |
|  | G306.1 | 30 | 22 | 15 |
|  | G306.2 | 30 | 29 | 26 |
|  | G308.65 | 31 | 22 | 22 |
|  | H3010.5 | 24 | 28 | 30 |
|  | I306.2 | 28 | 29 | 26 |
|  | H308.2 | 30 | 17 | 28 |
|  | I308.32 | 28 | 24 | 20 |
|  | G377.8 | 24 | 28 | 32 |
|  | G378.30 | 24 | 25 | 26 |
|  | D303.36 | 26 | 19 | 22 |
|  | H3010.1 | 26 | 27 | 33 |
|  | D371.5 | 19 | 25 | 29 |
|  | D372.20 | 18 | 24 | 24 |
|  | D373.37 | 18 | 19 | 25 |
|  | I379.8 | 22 | 29 | 24 |
| ***L. coryniformis*** | I306.12 | 11 | 14 | 19 |
|  | H307.1 | 29 | 20 | 28 |
|  | C305.1 | 7 | 11 | 12 |
|  | H307.6 | 15 | 20 | 13 |
|  | H376.2 | 9 | 6 | 19 |
|  | H376.5 | 9 | 14 | 9 |
|  | H377.3 | 25 | 21 | 24 |
| **Median value** | | 24.5 | 21.5 | 24 |
