# Supplementary material for: In Vitro and in Vivo Selection of Potentially Probiotic Lactobacilli From Nocellara del Belice Table Olives
Source: Front Microbiol. 2018 Mar 28;9:595. doi: 10.3389/fmicb.2018.00595 (PMC5882814; doi:10.3389/fmicb.2018.00595)
Supplement: Supplementary file 2 [file Presentation1.PPTX]

## Slide 1
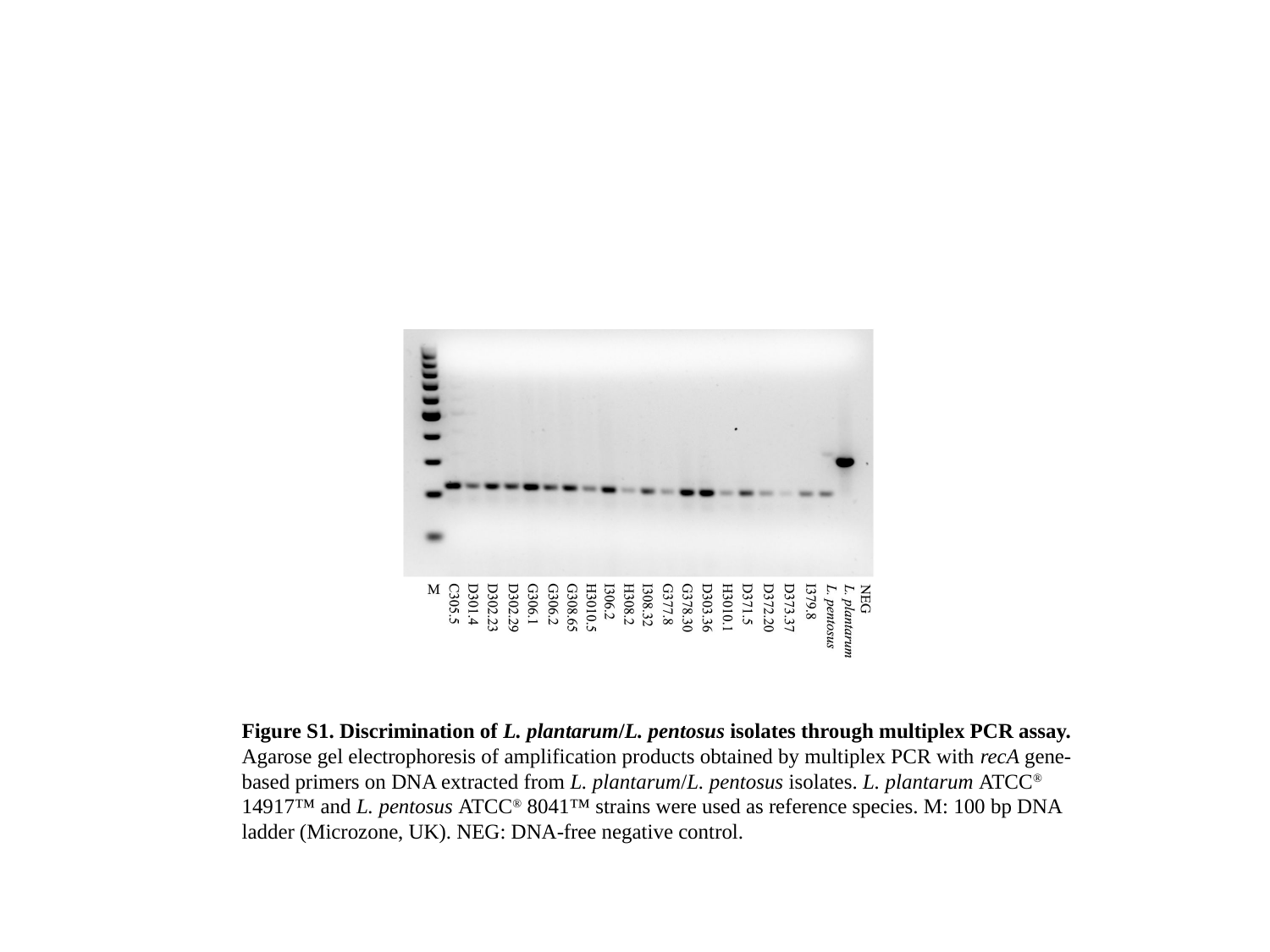

Figure S1. Discrimination of L. plantarum/L. pentosus isolates through multiplex PCR assay.
Agarose gel electrophoresis of amplification products obtained by multiplex PCR with recA gene-based primers on DNA extracted from L. plantarum/L. pentosus isolates. L. plantarum ATCC® 14917™ and L. pentosus ATCC® 8041™ strains were used as reference species. M: 100 bp DNA ladder (Microzone, UK). NEG: DNA-free negative control.
