# Supplementary material for: In Vitro and in Vivo Selection of Potentially Probiotic Lactobacilli From Nocellara del Belice Table Olives
Source: Front Microbiol. 2018 Mar 28;9:595. doi: 10.3389/fmicb.2018.00595 (PMC5882814; doi:10.3389/fmicb.2018.00595)
Supplement: Supplementary file 3 [file Presentation2.PPTX]

## Slide 1
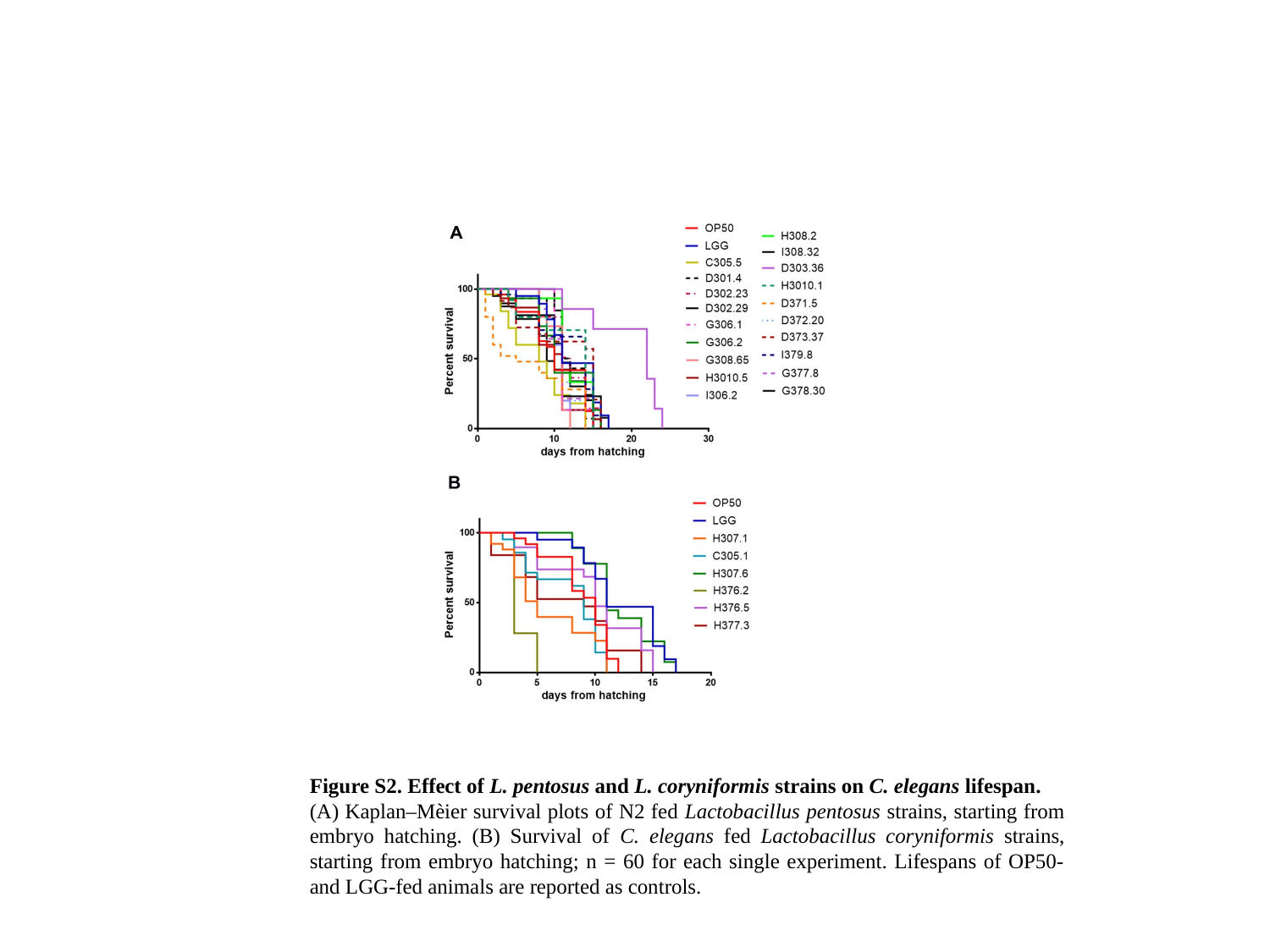

Figure S2. Effect of L. pentosus and L. coryniformis strains on C. elegans lifespan.
(A) Kaplan–Mèier survival plots of N2 fed Lactobacillus pentosus strains, starting from embryo hatching. (B) Survival of C. elegans fed Lactobacillus coryniformis strains, starting from embryo hatching; n = 60 for each single experiment. Lifespans of OP50- and LGG-fed animals are reported as controls.
